# Cardiac biomarkers in chronic kidney disease are independently associated with myocardial edema and diffuse fibrosis by cardiovascular magnetic resonance

**DOI:** 10.1186/s12968-021-00762-z

**Published:** 2021-06-07

**Authors:** Luca Arcari, Juergen Engel, Tilo Freiwald, Hui Zhou, Hafisyatul Zainal, Monika Gawor, Stefan Buettner, Helmut Geiger, Ingeborg Hauser, Eike Nagel, Valentina O. Puntmann

**Affiliations:** 1 Institute of Experimental and Translational Cardiovascular Imaging, DZHK Centre for Cardiovascular Imaging, Goethe University Hospital Frankfurt, Frankfurt am Main, Germany; 2 Cardiology Unit, Clinical and Molecular Medicine Department, Faculty of Medicine and Psychology, Sapienza University of Rome, Rome, Italy; 3 Department of Nephrology, Goethe University Hospital Frankfurt, Frankfurt am Main, Germany; 4 Department of Radiology, XiangYa Hospital, Central South University, Changsha, Hunan China; 5 Department of Cardiology, Universiti Teknologi MARA (UiTM), Sg. Buloh, Malaysia; 6 Department of Cardiology, University Hospital Warsaw, Warsaw, Poland

**Keywords:** Chronic kidney disease, Heart failure, Myocardial remodeling, Troponin, Edema, Cardiovascular magnetic resonance

## Abstract

**Background:**

High sensitivity cardiac troponin T (hs-cTnT) and NT-pro-brain natriuretic peptide (NT-pro BNP) are often elevated in chronic kidney disease (CKD) and associated with both cardiovascular remodeling and outcome. Relationship between these biomarkers and quantitative imaging measures of myocardial fibrosis and edema by T1 and T2 mapping remains unknown.

**Methods:**

Consecutive patients with established CKD and estimated glomerular filtration rate (eGFR) < 59 ml/min/1.73 m^2^ (n = 276) were compared to age/sex matched patients with eGFR ≥ 60 ml/min/1.73 m^2^ (n = 242) and healthy controls (n = 38). Comprehensive cardiovascular magnetic resonance (CMR) with native T1 and T2 mapping, myocardial ischemia and scar imaging was performed with venous sampling immediately prior to CMR.

**Results:**

Patients with CKD showed significant cardiac remodeling in comparison with both healthy individuals and non-CKD patients, including a stepwise increase of native T1 and T2 (p < 0.001 between all CKD stages). Native T1 and T2 were the sole imaging markers independently associated with worsening CKD in patients [B = 0.125 (95% CI 0.022–0.235) and B = 0.272 (95% CI 0.164–0.374) with p = 0.019 and < 0.001 respectively]. At univariable analysis, both hs-cTnT and NT-pro BNP significantly correlated with native T1 and T2 in groups with eGFR 30–59 ml/min/1.73 m^2^ and eGFR < 29 ml/min/1.73 m^2^ groups, with associations being stronger at lower eGFR (NT-pro BNP (log transformed, lg10): native T1 r = 0.43 and r = 0.57, native T2 r = 0.39 and r = 0.48 respectively; log-transformed hs-cTnT(lg10): native T1 r = 0.23 and r = 0.43, native T2 r = 0.38 and r = 0.58 respectively, p < 0.001 for all, p < 0.05 for interaction). On multivariable analyses, we found independent associations of native T1 with NT-pro BNP [(B = 0.308 (95% CI 0.129–0.407), p < 0.001 and B = 0.334 (95% CI 0.154–0.660), p = 0.002 for eGFR 30–59 ml/min/1.73 m^2^ and eGFR < 29 ml/min/1.73 m^2^, respectively] and of T2 with hs-cTnT [B = 0.417 (95% CI 0.219–0.650), p < 0.001 for eGFR < 29 ml/min/1.73 m^2^].

**Conclusions:**

We demonstrate independent associations between cardiac biomarkers with imaging markers of interstitial expansion, which are CKD-group specific. Our findings indicate the role of diffuse non-ischemic tissue processes, including excess of myocardial fluid in addition to diffuse fibrosis in CKD-related adverse remodeling.

**Supplementary Information:**

The online version contains supplementary material available at 10.1186/s12968-021-00762-z.

## Background

Chronic kidney disease (CKD) and heart failure (HF) often coexist and worsening renal function is a strong predictor of poor cardiovascular outcome [1]. Patients with CKD have very high risk of cardiovascular disease (CVD), yet high CVD morbidity and mortality is only partially explained by the complications from atherosclerotic coronary artery disease (CAD). Phenotypically, HF in CKD is characterized by preserved left ventricular (LV) systolic function, eccentric remodeling and increased diastolic stiffness [2–4]. Markedly raised troponin and N-terminal pro-brain natriuretic peptide (NT-pro BNP), the markers of myocardial injury and increased wall-stress, respectively, are also frequently found in CKD. Both markers are associated with poor prognosis in CKD, and especially with the HF-associated outcomes [5–8]. Although their significance remains controversially discussed and had been related to impaired renal elimination, recent reports imply that a sustained, subclinical, non-ischemic myocardial injury is more likely to explain the raised levels of these markers in CKD [9].

Myocardial T1 and T2 mapping with cardiovascular magnetic resonance (CMR) allows quantifiable tissue characterization and provide direct measures of the pathological myocardial processes non-invasively [10]. Native T1 is a non-specific measure of abnormal myocardium; it can relate to myocardial fibrosis, edema or infiltration [10]. Native T2 is water-specific, indicating excess myocardial fluid, which co-localizes with myocardial processes, such as edema, injury and/or inflammation [10]. Several studies revealed higher native T1 values in CKD patients [11], whereas more recently, a correlation of native T1 and troponin has also been shown [12]. A single previous study revealed raised T1 and T2 markers in participants with severe CKD on hemodialysis [13]. Native T1 of non-scarred myocardium is a strong predictor of survival, CVD mortality and incidence of HF in ischemic and non-ischemic cardiac conditions [14, 15]. No study to date comprehensively examined the relationship between these imaging measures and myocardial changes in CKD, nor related these to the serological markers of myocardial injury and increased wall stress, high-sensitive cardiac troponin T (hs-cTnT) and NT-pro BNP.

## Methods

**Fig. 1:**
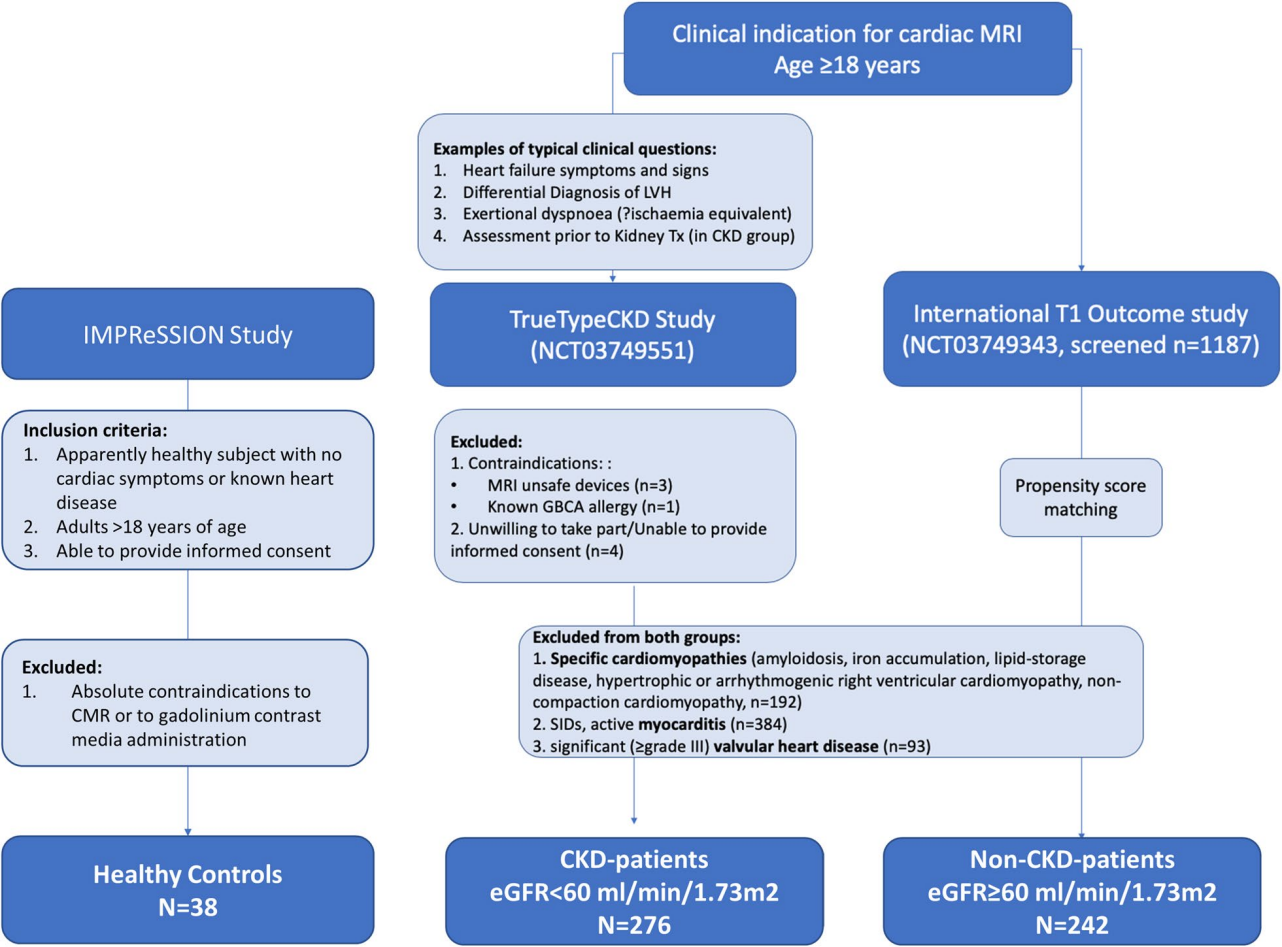
Consort diagram. Study details and imaging protocols available at the dedicated webpages: TrueTypeCKD Study and International T1 Outcome study. *CKD* chronic kidney disease, *GBCA* gadolinium-based contrast agent, *LVH* left ventricular hypertrophy, *MRI* magnetic resonance imaging, *SID* systemic inflammatory disease, *Tx* transplantation

This is a prospective longitudinal observational investigator-led study of T1 in adult patients undergoing a clinically indicated CMR examination, in line with cardiological practice guidelines (Consort diagram, Fig. 1). Consecutive patients with established diagnosis of CKD [16, 17] and reduced estimated glomerular filtration rate (eGFR) < 59 ml/min/1.73 m^2^ using the Modification of Diet in Renal Disease (MDRD) formula (the CKD stages 3–5; TRUE-TypeCKD Study NCT03749551) ongoing recruitment since March 2018) were enrolled. The CKD diagnosis was established independently by nephrologists and defined as abnormalities of kidney function or structure present for more than 3 months and eGFR < 59 ml/min/1.73 m^2^ on at least two occasions separated by a period of at least 90 days (with or without markers of kidney damage) [16, 17]. An independent cohort of controls with eGFR ≥ 60 ml/min/1.73 m^2^ (including CKD stages 1 and 2 [16, 17]), matched for age, gender and traditional CVD risk factor profile, was sourced from the ongoing International T1 Outcome Study using propensity score matching (PSM, n = 242, recruited between 03/16 to 03/19 (NCT03749343), ongoing recruitment since March 2016). The details of both registries, inclusion/exclusion criteria, imaging protocols and sequence parameters were reported previously [14, 15, 18], and are included in Additional file 3. A further group of healthy controls (n = 38) with similar age and sex distribution was gathered from an ongoing multicenter study including healthy individuals without CVD risk factors or any regular medication (NCT04444128). Exclusion criteria were known specific cardiomyopathies, valvular heart disease or myocarditis, known allergy to gadolinium-based contrast agents (GBCA), and CMR unsafe implants or devices. All procedures were carried out in accordance with the Declaration of Helsinki (2013). All participants were informed about the possible risks of nephrogenic systemic fibrosis (NSF), as per regulatory guidelines (Food and Drug Administration, European Medicine Agency, American College of Radiology [19]). Administration of GBCA to the CKD patients was in any case strictly performed following guidelines indication [20]. Patients on hemodialysis were scheduled to receive this within 24 h from the administration of the GBCA. All participants with significant CKD were screened for symptoms of NSF at regular 6-monthly intervals. The study protocols were reviewed and approved by institutional ethics committee. Written informed consent was obtained from all participants.

**Fig. 2:**
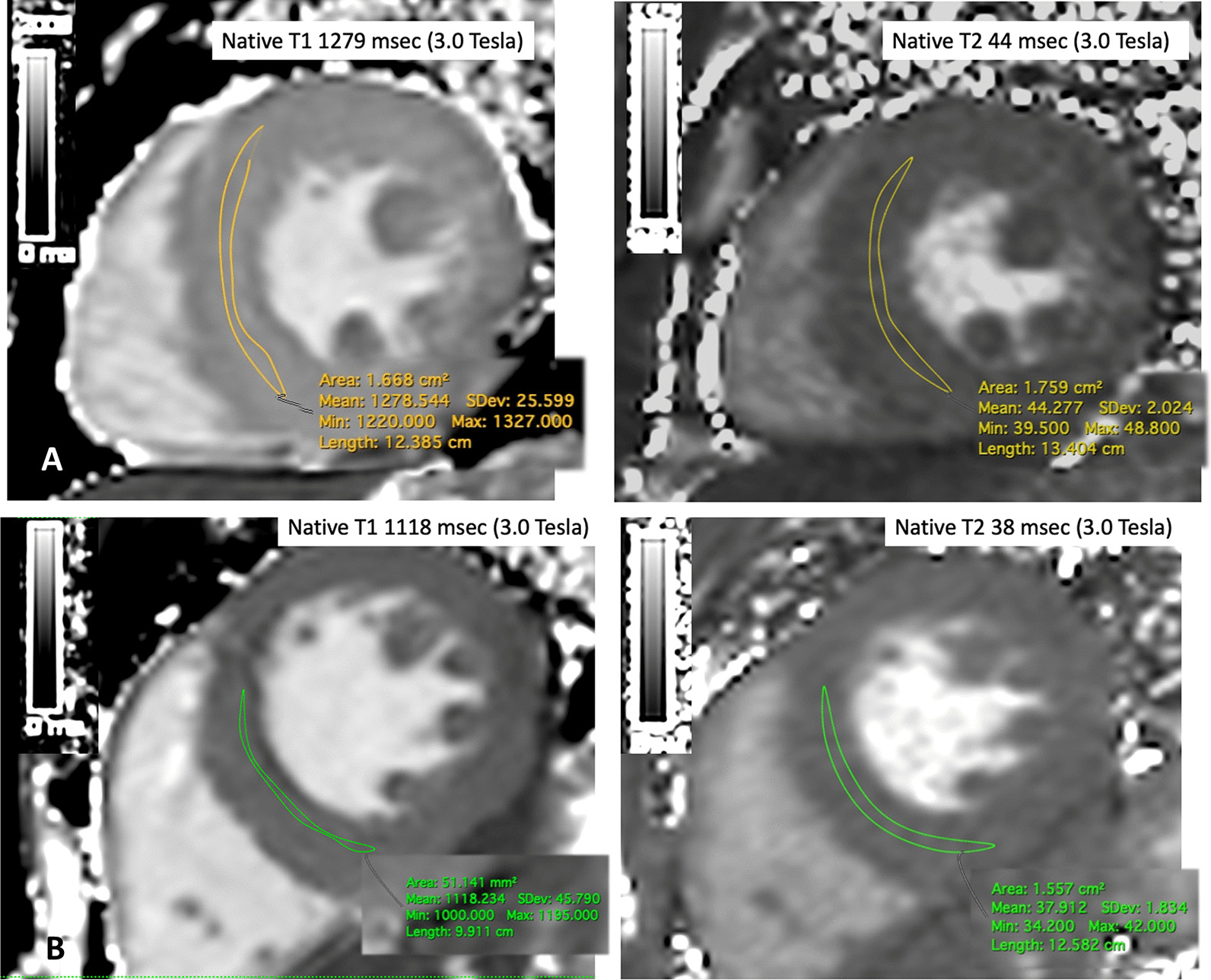
Representative measurement of native T1 and T2 in a CKD patient (**a**) and a control (**b**) Native T1 and T2 measurements (mean midventricular septal ROI measurement, normal values for native T1 using FFM-MOLLI: 3.0-T: mean of the normal range 1052 ± 23 ms; i.e. upper limit of normal range: 1098 ms at 3T), native T2: T2-FLASH sequence 35 ± 2 ms)

Clinical meta-data, including systolic/diastolic blood pressure (BP), body mass index (BMI), presence of traditional CVD risk factors, symptoms, medication were collected for all participants. Respiratory variation of inferior vena cava (IVC) from transthoracic echocardiography performed within 30 min from the index CMR was used as an index of fluid status, obtainable in all subjects. Loop diuretics use was not considered a reliable marker of volume status in CKD patients, since hemodialysis patients with no urine production would likely have volume overload and no diuretics intake. All participants underwent a standardized CMR using a 3T clinical CMR scanner (Skyra, software version VE11, Siemens Healthineers, Erlangen, Germany) for acquisition of cardiac function, volumes, mass, myocardial T1 and T2 mapping (Fig. 2), myocardial perfusion and scar imaging. T1 mapping was performed using an in-house developed variant of the modified Look-Locker Imaging sequence (Frankfurt Main, FFM-MOLLI) [10]. For T2 mapping, a T2-FLASH sequence was employed [21]. All T1 and T2 mapping values were determined by these same sequences, where both have established normal ranges, which were previously published using identical sequence parameters [22, 23]. Myocardial perfusion imaging was performed using vasodilation (regadenoson, 400 mcg/5 ml) and administration of 0.1 mmol/kg body weight gadobutrol (Gadovist^®^, Bayer Healthcare, Berlin, Germany) [24]. The presence of myocardial scar was visualized by late gadolinium enhancement (LGE) 15 min after GBCA administration.

Transthoracic echocardiography was performed by board-certified cardiologists within 30 min of completion of CMR examination with participants lying supine in left lateral position (Vivid E95, General Electric Healthcare, Chicago, Illinois, USA) for respiratory variation of IVC, as a measure of volume status, using postprocessing recommendation [25].

Analysis of cardiac volumes, function and mass was performed using semiautomated contour detection (SuiteHeart^®^, Neosoft, Pewaukee, Wisconsin, USA). Interpretation of myocardial perfusion and LGE images was performed following standardized postprocessing recommendations. Myocardial LGE was visually defined by a minimum of two observers based on the presence and predominant pattern as ischemic or non-ischemic [26]. All observers were board-certified cardiologists holding established accreditations of CMR competencies. Quantitative tissue characterization and myocardial deformation analysis were performed by the core-lab staff (Goethe CVI, Frankfurt/Main, Germany), blind to the underlying participant group allocation. Myocardial T1 and T2 were measured conservatively within septal myocardium of midventricular short axis slice, using motion-corrected scanner-derived images, as per internal standardized operating procedures [10, 27]. Areas of LGE were excluded from region of interest to avoid inclusion of areas with replacement scar. Global longitudinal (GLS) and circumferential strain (GCS) were measured using CMR feature tracking (MEDIS^®^, Leiden, The Netherlands) [28].

All participants underwent venous blood sampling immediately prior to CMR study. Bloods samples were spun and frozen at − 80 °C and analyzed subsequently using standardized commercially available test kits Analysis hs-cTnT and NT-pro BNP (Elecsys 2010^®^, Roche, Basel, Switzerland). The cut-offs for hs-cTnT and NT-pro BNP were used to define normal/abnormal in all subjects (using a cut of value of 99 percentile of 13.9 ng/l [8] and > 300 pg/l [3], respectively).

### Statistical analysis

Staistical analysis was performed using SPSS (version 25.0, Statistical Package for the Social Sciences, International Business Machines, Inc., Armonk, New York, USA and R Plug-in for PSM). Data are presented in counts (percentages), mean ± standard deviation (SD) or median (interquartile range, IQR), as appropriate. Group comparisons were performed using paired and independent t-test or one-way ANOVA, Chi^2^, Mann–Whitney test, and Fischer’s exact tests as appropriate. Correlation analysis was performed (graphically reported) to assess relationships between serological and imaging markers at various degrees of renal function. The interaction of imaging markers with status of CKD in patients (eGFR ≥ 90 ml/min/1.73 m^2^, eGFR 60–89 ml/min/1.73 m^2^, eGFR 30–59 ml/min/1.73 m^2^, eGFR < 29 ml/min/1.73 m^2^) was examined using univariable and multivariable linear regression. Relationships within the CKD strata were tested for interaction. The associations between serological and imaging measures were explored using by univarible and multivariable linear regressions. Since serum biomarkers displayed a highly skewed distribution, log-transformed values were used to achieve approximate normal distribution when performing regression analysis. Analysis of collinearity was performed by correlation matrix, where a correlation of ± 0.7 or weaker was used as cut-off for inclusion of variables within the same multivariable model. This criterion was not satisfied by LV end-diastolic volume (LVEDV) and LV end-systolic volume (LVESV) that showed closer correlation; hence, LVEDV only was included in the multivariable models. We included in the multivariable models those variables that had p < 0.05 at univariable analysis, plus all those having biological plausibility. Multivariable analysis was performed by stepwise regression. We estimated that a sample size of 37 per group was needed to achieve 80% power to detect continuous associations with a coefficient of determination r^2^ = 0.20, using a 2-sided hypothesis test with a significance level of 0.05. PSM was performed using 1:k optimal matching algorithm based on the following variables: traditional CVD risk factors (age, sex, presence of hypertension, diabetes, smoking, hyperlipidemia). Reproducibility of CMR measurements were assessed using Bland–Altman analyses. All tests were two-tailed and p-value of < 0.05 was considered statistically significant.

## Results

**Fig. 3:**
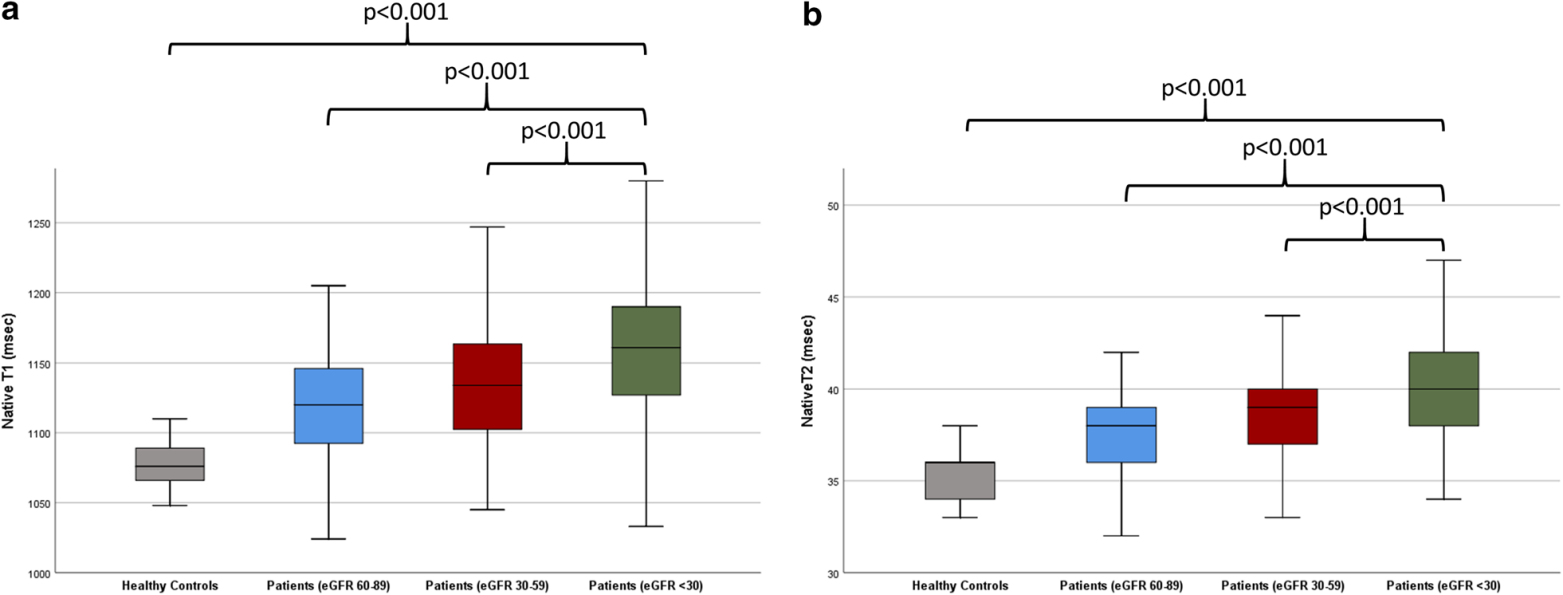
Native T1 and T2 values across groups of healthy controls and patients with differential degree of CKD

Baseline population characteristics for groups of patients and healthy controls are summarized in Table 1. Significant differences were observed between healthy controls and both non-CKD (eGFR ≥ 60 ml/min/1.73 m^2^) and CKD (eGFR < 59 ml/min/1.73 m^2^) patients’ groups regarding native T1 and T2 which were lower in controls (Table 2, Fig. 3 p < 0.001 for all comparisons). Compared to patients with eGFR ≥ 60 ml/min/1.73 m^2^, patients with eGFR < 59 ml/min/1.73 m^2^ had higher hs-cTnT, NT-pro BNP and lower hematocrit (p < 0.05). The two groups were similar for New York Heart Association class, and most cardiac medications, apart from the higher rate of aldosterone and neprilysin inhibitors in controls, and loop diuretics in the eGFR < 59 ml/min/1.73 m^2^ group (p < 0.05 for all). The eGFR < 59 ml/min/1.73 m^2^ group also had higher LV volumes and mass, myocardial native T1 and T2 (Fig. 3), and lower LV ejection fraction (LVEF), GCS and IVC respiratory variation (Table 2, p < 0.01 for all). A third of all patients had myocardial scar by LGE with no overall difference in proportions between the patients’ groups for the presence (p = 0.15) or patterns (ischemic vs. non-ischemic, p = 0.63 vs. 0.37, respectively). Patient groups were also similar for the presence of relevant myocardial ischemia (p = 0.10); this was treated with revascularization, if amenable target vessel was identified.

**Table 1:** Participant characteristics

Variable	Healthy controls (n = 38)	Patients (eGFR ≥ 60) (n = 242)	Patients (eGFR < 59) (n = 276)	Sig (p-value)*
Age (years)	57 ± 11	56 ± 19	58 ± 21	0.13
Male (n, %)	24 (63)	145 (60)	189(65)	0.24
Systolic BP (mmHg)	121 ± 11°	134 ± 17	137 ± 21	0.38
Diastolic BP (mmHg)	75 ± 6°	79 ± 10	78 ± 12	0.31
Heart rate (bpm)	64 ± 10	73 ± 13	75 ± 14	0.29
Blood hematocrit (%)	42 ± 4§	41.3 ± 5.2	39.8 ± 6.4	**0.004**
eGFR (ml/min/1.73 m^2^)	87(83–94)°	84(61–112)	29(6–57)	**<** **0.001**
hs-CRP, mg/l	0.2(0.1–0.3)°	3.9 ± 0.9	6.3 ± 1.8	**<** **0.001**
hs-cTnT (ng/l)	1(0–2)°	6 (4–10)	14 (6–30)	**0.01**
NT-pro BNP (pg/l)	48 (38–72)°	78 (38–207)	582 (187–2192)	**<** **0.001**
> 300, n (%)	/	46 (24)	69 (62)	**<** **0.001**
NYHA ≥ III (n, %)	/	68 (28)	88 (32)	0.32
BMI (kg/m^2^)	23 ± 2	27 ± 8	26 ± 9	0.185
Blood hemoglobin (g/dl)	14 ± 1§	14.2 ± 1	12.6 ± 1	**<** **0.001**
Smoking (n, %)	/	48 (20)	66 (24)	0.274
Hypertension (n, %)	/	192 (91)	262 (95)	0.073
Diabetes (n, %)	/	116 (48)	143 (52)	0.364
Type II (n, %)	/	87 (36)	112 (41)	0.244
Vasculitis (n, %)	/	39(16)	52(19)	0.546
Polycystic syndrome (n, %)	/	7(2)	12(4)	0.188
Hyperlipidemia (n, %)	/	150 (62)	188 (68)	0.153
Known CAD (n, %)	/	68 (28)	88 (32)	0.322
3-vessel CAD or equivalent (n, %)	/	32 (13)	48 (17)	0.205
Previous revascularization (n, %)	/	53 (22)	77 (28)	0.117
Previous diagnosis of HF (n, %)	/	77 (32)	108 (39)	0.097
Cardiac medication				
Beta blockers, n (%)	/	138(57)	174 (63)	0.299
RAS inhibitors, n (%)	/	198(82)	234(85)	0.358
Aldosterone inhibitors (n, %)	/	68(28)	33(12)	**<** **0.001**
Neprilysin inhibitors (n, %)	/	27(11)	2(5)	**0.011**
Calcium antagonists (n, %)	/	184(76)	224(81)	0.166
Loop diuretics (n, %)	/	68(28)	199(72)	**<** **0.001**
Platelet inhibition (n, %)	/	138(57)	136(51)	0.172
Statins (n, %)	/	155(64)	196(71)	0.089

Bold indicated p-value < 0.05

Mean ± SD, or median (IQR), p-value < 0.05 was considered significant

*BP* blood pressure, *eGFR* estimated glomerular filtration rate (ml/min/1.73 m^2^), *hs-cTnT* high-sensitive troponin T, *CAD* coronary artery disease, *CRP* C-reactive protein, *HF* heart failure, *NT-proBNP* N-terminal pro brain natriuretic peptide, *NYHA* New York Heart Association, *RAS* renin-angiotensin system

*p-value for differences between patients’ groups (eGFR ≥ 60 vs eGFR < 59)

°p < 0.05 vs non-CKD and CKD patients’ groups

^§^p < 0.05 vs CKD patients’ group

**Table 2:** Cardiovascular magnetic resonance (CMR) and echocardiographic measurements of function, structure and tissue characterization

Variable	Healthy controls (n = 38)	Patients (eGFR ≥ 60 ml/min/1.73 m^2^) (n = 242)	Patients (eGFR < 59 ml/min/1.73 m^2^) (n = 276)	Sig (p-value)*
LVEDV index, ml/m^2^	80 ± 11°	84 ± 20	93 ± 30	**<** **0.001**
LVESV index, ml/m^2^	31 ± 5°	38 ± 19	46 ± 31	**<** **0.001**
LVEF, %	61 ± 5°	57 ± 11	53 ± 17	**<** **0.001**
LV mass index, g/m^2^	65 ± 15§	63 ± 16	70 ± 21	**<** **0.001**
RVEF, %	62 ± 8°	57 ± 9	56 ± 13	0.42
LA area, cm^2^	21 ± 2°	23 ± 5	27 ± 7	**0.002**
GLS, %	24 ± 7§	24 ± 5	21 ± 8	**<** **0.001**
GCS, %	24 ± 3°	29 ± 4	27 ± 8	0.08
Myocardial LGE, n (%)	0 (0)°	70 (29)	97 (35)	0.15
Ischemic type, n (%)	0 (0)°	34 (14)	44 (16)	0.64
Non-ischemic, n (%)	0 (0)°	36 (15)	53 (19)	0.37
Myocardial ischemia, n (%)	0 (0)°	27 (11)	44 (16)	0.10
Native T1 (ms)	1076 ± 18°	1123 ± 31	1152 ± 43	**<** **0.001**
Native T1 > 1167 ms (5SD cut-off) (%)	/	24 (10)	83 (34)	**<** **0.001**
Native T2 (ms)	35 ± 1°	37 ± 2	41 ± 4	**<** **0.001**
Native T2 > 40 ms (2SD cut-off) (%)	/	43 (17)	117 (46)	**<** **0.001**
Echo parameters				
IVC diameter (mm)	10.3 ± 4°	9.8 ± 4.6	15.9 ± 3.1	**<** **0.001**
IVC change (insp) (%)	57 ± 6§	58 ± 14	19 ± 23	**<** **0.001**

Bold indicated p-value < 0.05

Mean ± SD, or median (IQR), p-value < 0.05 was considered significant

*eGFR* estimated glomerular filtration rate (in units, ml/min/1.73 m^2^), *EDV* end-diastolic volume, *ESV* end-systolic volume, *EF* ejection fraction, *GLS* global longitudinal strain, *GCS* global circumferential strain, *LV* left ventricular, *LGE* late gadolinium enhancement, *IVC* inferior vena cava, *SD* standard deviation

^*^p-value for comparison between patients’ groups (eGFR ≥ 60 vs eGFR < 59)

°p < 0.05 vs non-CKD and CKD patients’ groups

^§^p < 0.05 vs CKD patients’ group

### Pre and post-hemodialysis comparisons

**Fig. 4:**
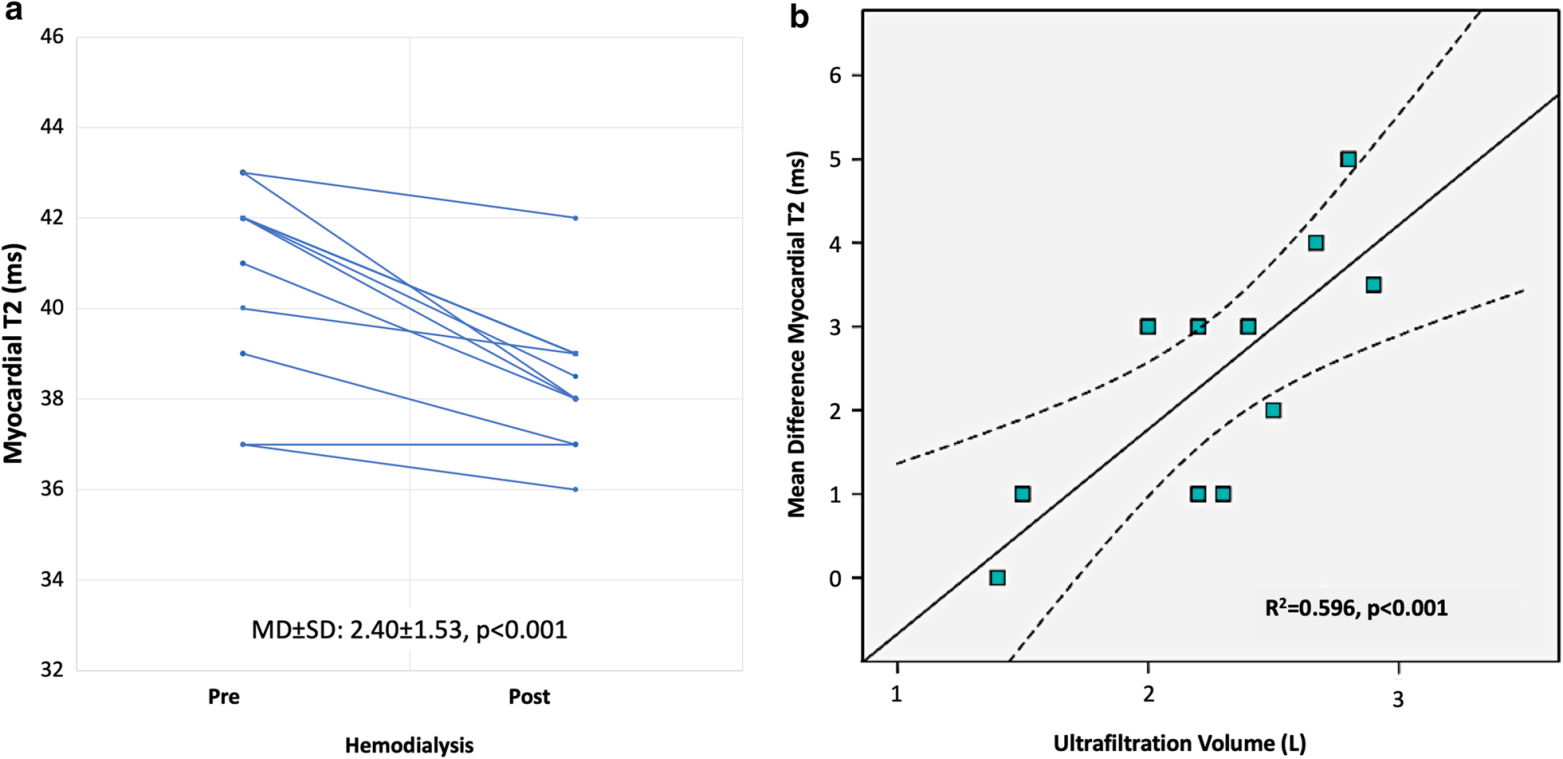
Subgroup of participants undergoing a repeat CMR immediately after hemodialysis. **a** Graph showing significant native T2 reduction after hemodialysis. **b** Graph showing significant correlation between removed ultrafiltration volume and change in native T2. *MD* mean difference, *SD* standard deviation

A total of 10 participants with severe CKD on HD underwent a repeat native CMR (Fig. 4) immediately after completed HD. There was a reduction of native T2 (mean difference ± standard deviation = 2.40 ± 1.53 ms, p < 0.001), LVEDV (4.0 ± 1.4 ml, p < 0.001), and smaller IVC diameter (3.1 ± 2.4 mm, p < 0.001), but no significant change in LV mass (1.9 ± 1.2 g, p = 0.09). The mean difference in myocardial T2, LVEDV and LV mass were all proportional to the amount of removed ultrafiltration volume (r = 0.72, r = 0.65 and r = 0.41 respectively, p < 0.001 for all).

### Analysis of relationships

**Fig. 5:**
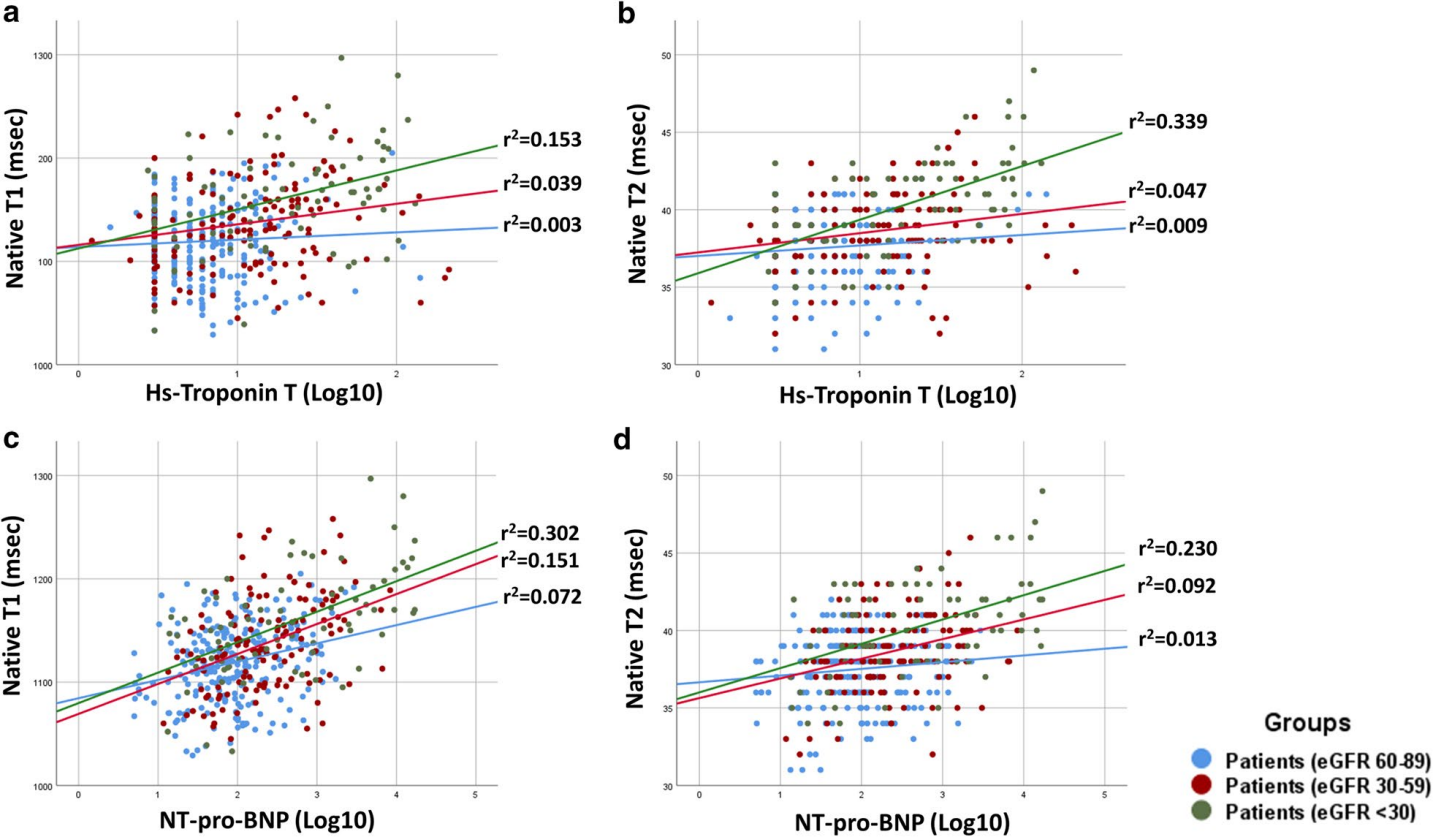
Relationships between high sensitivity cardiac troponin Ths-cTnT (**a**, **b**) and N-terminal pro brain natriuretic peptide (NT-pro BNP) (**c**, **d**) with native T1 and T2 in patients with differential degrees of CKD. *hs-cTnT* high-sensitive cardiac troponin T, *lg10* log transformed

Subgroups stratified by eGFR showed differentially significant associations between hs-cTnT and native T1 and T2 (Fig. 5, Additional file 1: Figure S1 and Additional file 2: Figure S2). Overall, the relationships between the two tissue imaging markers, serological biomarkers and measures of structural remodeling were stronger in the eGFR < 59 ml/min/1.73 m^2^ group. Also, there was a stronger inter-relationship between native T1 and T2 as eGFR declined (eGFR ≥ 60 ml/min/1.73 m^2^ vs. eGFR 30–59 ml/min/1.73 m^2^ vs. eGFR < 29 ml/min/1.73 m^2^, r = 0.295. vs r = 0.607 vs r = 0.560, p < 0.001 for all, p < 0.05 for interaction). Both hs-cTnT and NT-pro BNP were significantly associated with native T1 and T2 in groups with eGFR 30–59 ml/min/1.73 m^2^ and eGFR < 29 ml/min/1.73 m^2^ groups, with the association being stronger at the lower eGFR (log-transformed NT-pro BNP: native T1 r = 0.43 and r = 0.57, native T2 r = 0.39 and r = 0.48 respectively; log-transformed hs-cTnT: native T1 r = 0.23 and r = 0.43, native T2 r = 0.38 and r = 0.58 respectively, p < 0.001 for all, p < 0.05 for interaction); a weaker relationship was observed between hs-cTnT and native T1 and T2 in patients with eGFR 60–89 ml/min/1.73 m^2^ (r = 0.18 and r = 0.19 with p = 0.004 and p = 0.003 respectively). IVC change during inspiration showed a negative correlation with both native T1 and T2, albeit only in patients with eGFR < 29 ml/min/1.73 m^2^ (r = − 0.33 and r = − 0.36, with p = 0.003 and p = 0.001 respectively).

In adjusted linear regression analyses, native T1 and T2 were independently associated with the status of worsening CKD [B = 0.125 (95% CI 0.022–0.235) and B = 0.272 (95% CI 0.164–0.374), respectively] (Table 3). Multivariable linear analyses (stepwise, forward, likelihood-ratio, including as independent variables also CMR measures of LV function, mass and scar as well as IVC respiratory variation as a measure of volume status, hematocrit and C-reactive protein concentration) revealed independent associations of NT-pro BNP with native T1 in patients with eGFR < 59 ml/min/1.73 m^2^ only [eGFR 30–59 ml/min/1.73 m^2^: B = 0.308 (95% CI 0.129–0.407), p < 0.001; eGFR < 29 ml/min/1.73 m^2^: B = 0.334 (95% CI 0.154–0.660), p = 0.002] and of hs-cTnT with native T2 in eGFR < 29 ml/min/1.73 m^2^ only [B = 0.417 (95% CI 0.219–0.650), p < 0.001] (Table 4).

**Table 3:** Univariable and multivariable linear regression analysis for factors associated with worsening CKD

Variable	Worsening CKD			
	Univariable		Multivariable	
	B (95% CI)	p-value	B (95% CI)	p-value
Hematocrit (%)	− 0.193 (− 0.283 to − 0.103)	**<** **0.001**	NS	NS
hs-CRP (Lg10)	0.085 (− 0.006 to 0.177)	0.068	0.098 (0.009 to 0.185)	**0.031**
LVEDV index (ml/m^2^)	0.204 (0.114 to 0.293)	**<** **0.001**	NS	NS
LV mass index (g/m^2^)	0.208 (0.119 to 0.297)	**<** **0.001**	NS	NS
LVEF (LVEF)	− 0.068 (− 0.159 to 0.023)	0.143	NS	NS
GLS (%)	0.041 (− 0.050 to 0.133)	0.376	NS	NS
LA area (cm^2^)	0.153 (0.054 to 0.251)	**0.002**	NS	NS
IVC variation (%)	− 0.341 (− 0.427 to − 0.255)	**<** **0.001**	− 0.242 (− 0.357 to − 0.158)	**<** **0.001**
LGE (presence)	0.024 (− 0.067 to 0.116)	0.600	NS	NS
Native T1 (ms)	0.348 (0.263 to 0.434)	**<** **0.001**	0.125 (0.022 to 0.235)	**0.019**
Native T2 (ms)	0.385 (0.301 to 0.470)	**<** **0.001**	0.272 (0.164 to 0.374)	**<** **0.001**

Bold indicated p-value < 0.05

*eGFR* estimated glomerular filtration rate (in units, ml/min/1.73 m^2^), *CI* confidence interval, *CRP* C-reactive protein, *EV* end-diastolic volume, *EF* ejection fraction, *GLS* global longitudinal strain, *LV* left ventricular, *LGE* late gadolinium enhancement, *IVC* inferior vena cava

P-value < 0.05 was considered significant

**Table 4:** Univariable and multivariable linear regression analysis for factors associated with serological cardiac biomarkers according to different CKD stages

Variable	eGFR ≥ 60 ml/min/1.73 m^2^							
	hs-cTnT (Lg10)				NT-pro BNP (Lg10)			
	Univariable		Multivariable		Univariable		Multivariable	
	B (95% CI)	p-value	B (95% CI)	p-value	B (95% CI)	p-value	B (95% CI)	p-value
Hematocrit (%)	− 0.015 (− 0.106 to 0.083)	0.816	NS	NS	− 0.189 (− 0.263 to − 0.052)	**0.004**	− 0.233 (− 0.289 to − 0.099)	**<** **0.001**
hs-CRP (Lg10)	0.062 (− 0.049 to 0.139)	0.344	NS	NS	0.133 (0.005 to 0.216)	**0.041**	NS	NS
LVEDV (ml/m^2^)	0.255 (0.108 to 0.315)	**<** **0.001**	0.166 (0.034 to 0.242)	0.1	0.337 (0.204 to 0.433)	**<** **0.001**	NS	NS
LV mass index (g/m^2^)	0.154 (0.022 to 0.225)	**0.018**	NS	NS	0.135 (0.007 to 0.239)	0.038	NS	NS
LVEF (%)	− 0.253 (− 0.299 to − 0.102)	**0.001**	NS	NS	− 0.427 (− 0.490 to − 0.280)	**<** **0.001**	− 0.398 (− 0.465 to − 0.254)	**<** **0.001**
GLS (%)	0.221 (0.069 to 0.251)	**0.001**	NS	NS	0.345 (0.15 to 0.385)	**<** **0.001**	NS	NS
LA (cm^2^)	0.18 (0.038 to 0.238)	**0.007**	NS	NS	0.298 (0.149 to 0.369)	**<** **0.001**	0.208 (0.079 to 0.282)	**0.001**
IVC variation (%)	0.108 (− 0.02 to 0.231)	**0.099**	NS	NS	− 0.015 (− 0.160 to 0.126)	0.815	NS	NS
LGE (presence)	0.387 (0.189 to 0.356)	**<** **0.001**	0.345 (0.155 to 0.331)	**<** **0.001**	0.291 (0.135 to 0.332)	**<** **0.001**	NS	NS
Native T1 (ms)	0.059 (− 0.060 to 0.157)	0.368	NS	NS	0.269 (0.140 to 0.378)	**<** **0.001**	NS	NS
Native T2 (ms)	0.096 (− 0.028 to 0.193)	0.141	NS	NS	0.114 (− 0.013 to 0.237)	0.08	NS	NS

Variable	eGFR: 30–59							
Hematocrit (%)	− 0.097 (− 0.277 to 0.074)	0.256	NS	NS	− 0.165 (− 0.290 to 0.003)	0.055	NS	NS
hs-CRP (Lg10)	0.255 (0.090 to 0.416)	**0.003**	NS	NS	0.389 (0.190 to 0.451)	**<** **0.001**	NS	NS
LVEDV (ml/ m^2^)	0.207 (0.037 to 0.324)	**0.014**	NS	NS	0.381 (0.161 to 0.390)	**<** **0.001**	NS	NS
LV mass index (g/m^2)^	0.291 (0.133 to 0.463)	**<** **0.001**	0.218 (0.018 to 0.428)	**<** **0.001**	0.308 (0.123 to 0.400)	**<** **0.001**	NS	NS
LVEF (%)	− 0.271 (− 0.381 to − 0.096)	**0.001**	NS	NS	− 0.417 (− 0.419 to − 0.191)	**<** **0.001**	NS	NS
GLS (%)	0.281 (0.119 to 0.444)	**0.001**	NS	NS	0.467 (0.260 to 0.514)	**<** **0.001**	0.319 (0.117 to 0.405)	**<** **0.001**
LA (cm^2^)	0.307 (0.098 to 0.423)	**0.002**	0.242 (0.035 to 0.375)	**0.002**	0.500 (0.227 to 0.477)	**<** **0.001**	0.311 (0.095 to 0.343)	**0.001**
IVC variation (%)	0.02 (− 0.222 to 0.282)	0.813	NS	NS	− 0.065 (− 0.293 to 0.132)	0.454	NS	NS
LGE (presence)	0.256 (0.087 to 0.394)	**0.002**	NS	NS	0.368 (0.162 to 0.412)	**<** **0.001**	NS	NS
Native T1 (ms)	0.189 (0.033 to 0.379)	**0.025**	NS	NS	0.389 (0.201 to 0.475)	**<** **0.001**	0.308 (0.129 to 0.407)	**<** **0.001**
Native T2 (ms)	0.217 (0.057 to 0.410)	**0.01**	NS	NS	0.304 (0.126 to 0.418)	**<** **0.001**	NS	NS

Variable	eGFR < 29							
Hematocrit (%)	− 0.5 (− 0.73 to − 0.318)	**<** **0.001**	− 0.307 (− 0.537 to − 0.107)	**0.004**	− 0.42 (− 0.736 to − 0.239)	**<** **0.001**	− 0.303 (− 0.575 to − 0.128)	**0.003**
hs-CRP (Lg10)	0.168 (− 0.061 to 0.432)	0.139	NS	NS	0.167 (− 0.080 to 0.489)	**0.157**	NS	NS
LVEDV (ml/m^2^)	0.369 (0.160 to 0.586)	**0.001**	NS	NS	0.497 (0.327 to 0.786)	**<** **0.001**	NS	NS
LV mass index (g/m^2^)	0.389 (0.165 to 0.546)	**<** **0.001**	NS	NS	0.575 (0.385 to 0.776)	**<** **0.001**	0.390 (0.183 to 0.604)	**0.001**
LVEF (%)	− 0.135 (− 0.368 to 0.091)	0.234	NS	NS	− 0.305 (− 0.599 to 0.090)	**0.009**	NS	NS
GLS (%)	0.251 (0.037 to 0.548)	**0.026**	NS	NS	0.401 (0.237 to 0.796)	**<** **0.001**	NS	NS
LA (cm^2^)	0.407 (0.179 to 0.707)	**0.001**	0.284 (0.097 to 0.522)	**0.005**	0.400 (0.181 to 0.784)	**0.002**	NS	NS
IVC variation (%)	− 0.106 (− 0.275 to 0.100)	0.354	NS	NS	− 0.237 (− 0.430 to − 0.007)	**0.043**	NS	NS
LGE (presence)	0.174 (− 0.064 to 0.506)	0.127	NS	NS	0.310 (0.119 to 0.750)	**0.008**	NS	NS
Native T1 (ms)	0.388 (0.189–0.657)	**<** **0.001**	NS	NS	0.549 (0.423 to 0.904)	**<** **0.001**	0.334 (0.154 to 0.660)	**0.002**
Native T2 (ms)	0.583 (0.415–0.799)	**<** **0.001**	0.417 (0.219 to 0.650)	**<** **0.001**	0.480 (0.314 to 0.793)	**<** **0.001**	NS	NS

Bold indicated p-value < 0.05

*eGFR* estimated glomerular filtration rate (in units, ml/min/1.73 m^2^), *CI* confidence interval, *CRP* C-reactive protein, *EV* end-diastolic volume, *ESV* end-systolic volume, *EF* ejection fraction, *GLS* global longitudinal strain, *LV* left ventricular, *LGE* late gadolinium enhancement, *IVC* inferior vena cava

p-value < 0.05 was considered significant

### Reproducibility of measurements

T1 and T2 mapping showed excellent intra- and inter-observer agreement: native T1: intra-observer: r = 0.98, p < 0.01, mean difference (MD) ± SD − 0.2 ± 5.3 ms; interobserver: r = 0.96, p < 0.01, MD ± SD − 2.4 ± 10.3 ms. T2 mapping: intraobserver: r = 0.98; MD ± SD = − 1.2 ± 1.5; interobserver r = 0.96, p < 0.001 MD ± SD = 1.8 ± 3.3). GLS and GCS strain analysis, intra- and inter-observer agreements were moderate: GLS: intra-observer: r = 0.78, p < 0.001, MD ± SD of 0.1 ± 3.1, interobserver: r = 0.72, 1.0 ± 4.3.

## Discussion

In this prospective study, we demonstrate independent associations between cardiac biomarkers with imaging marker of myocardial edema and diffuse fibrosis, which are CKD-group specific. Native T1 and T2 were the only imaging markers to be independently associated with worsening CKD. Serological marker of increased LV wall stress, NT-pro BNP, and of myocardial injury, hs-cTnT, were independently associated with native T1 and T2 respectively, albeit only in patients with reduced renal function. Also, there was a significant reduction of native T2 when measured immediately post-hemodialysis with the change in T2 proportional to the removed ultrafiltration volume. Together, these results indicate a link between myocardial injury and LV wall stress with an increase of myocardial fluid, in addition to myocardial fibrosis, which is inherent to the presence of CKD.

Our observations expand the current knowledge about the pathophysiology of CVD and adverse myocardial remodeling in CKD. We employed two independent approaches of detecting myocardial abnormalities, by serology and imaging, which are both well established in terms of accuracy and prognostic significance [3, 8, 10]. Previous studies investigating the causes of raised troponin in CKD indicated that increased levels cannot be explained by the classical myocardial injury processes, such as such as infarction-like necrosis. Our findings lend support to this notion by showing that despite the higher CVD risk given by renal insufficiency, the rate of ischemic scar and myocardial ischemia or infarction was similar to that of the non-CKD group. Compared to a matched cohort of patients with eGFR ≥ 60 ml/min/1.73 m^2^, but similar CVD risk factors by PSM of the two cohorts, we report elevated native T1 and T2, but not more prevalent LGE, reiterating that markedly raised troponin levels are unlikely fully explained by the consequences of atherosclerotic CAD [8]. Furthermore, we found that serological biomarkers hs-cTnT and NT-pro BNP and imaging markers of structural remodeling have closer correlation with native T1 and T2 as the renal function progressively declined. Together, these findings indicate that non-ischemic processes play an important role in pathophysiology of myocardial injury in CKD [29].

The independent association between native T2 and hs-cTnT in patients with severe CKD (eGFR < 29 ml/min/1.73 m^2^) is a central new finding, reiterating the role of increased myocardial fluid, in addition to myocardial fibrosis, as an integral part of structural LV remodeling in CKD [30]. In the literature, troponin release in CKD has been attributed to a number of mechanisms, including increased transmural pressure, small-vessel coronary obstruction, endothelial dysfunction, intracellular edema [8, 31, 32], as well as direct cellular toxicity of the uremic milieu [33, 34]. Notably, we strived to control for myocardial inflammation by excluding subjects with suspected clinical presentation or positive histology for active inflammation, which could yield a similar imaging pattern [35]. Whether edema is a cause or a consequence of the myocardial damage, cannot be fully elucidated based on the current data. Recent studies revealed the dynamic changes in native T2 measurements induced by either ultrafiltration or diuretics in CKD and HF, respectively [13, 36]. In a proof-of concept sub study, we reproduced this finding in a subgroup of participants that underwent a second scan immediately after HD. In addition to reduction of native T2 in this subgroup, we also found a trend of increasing association between native T2, hs-cTnT and NT-pro BNP with worsening of CKD stages, which may suggest that myocardial water content is increasing with worsening renal function, likely affected by the total body water content. This observation might point towards potential clinical implications. Despite discrete native T1 and T2 values overlap between non-CKD (eGFR ≥ 60 ml/min/1.73 m^2^) and CKD (eGFR < 59 ml/min/1.73 m^2^) patients, which could reduce the added value of mapping assessment in this context, T2 exhibited significant correlation with cardiac injury in patients with severe renal insufficiency (eGFR < 29 ml/min/1.73 m^2^) only. Further studies are required to explore whether focused myocardial water-shifts might translate into a modifiable intervention conferring cardio-protection in some patients with advanced CKD, hypothetically those with higher T2 and hs-cTnT. We also observed an interrelatedness between T1 and T2, which becomes much stronger as renal function declines, indirectly supporting that both measures are much more influenced by excess myocardial fluid in CKD [10, 27]. Accordingly, native T1 is a nonspecific measure of interstitial expansion, detecting water as well as fibrosis [30], hence, an integrated reading along with T2 values and a patient’s underlying renal function may aid the correct interpretation of this measure. Outcome data are needed to validate the prognostic relevance of our findings, as well as prospective trials of intervention algorithms based on the presented imaging readouts to ascertain whether their guidance can improve clinical care.

## Limitations

A few limitations of our study apply. We strived to control for inclusion bias in several ways. Respiratory variation of IVC was used as a proxy-measure of the total body fluid status, because it was obtainable in all subjects across the eGFR spectrum. Participants with reduced kidney function were recruited from tertiary centers as well as peripheral nephrology practices, thus reducing the potential referral bias. The clinical indications for CMR were uniform for both groups and approved by an independent cardiologist in line with cardiological practice guidelines. Native T1 moderately correlates with collagen volume fraction in model diseases of pressure overload (severe aortic stenosis, participants with eGFR ≥ 60 ml/min/1.73 m^2^), however, histological correlations with myocardial fibrosis in CKD may differ and require verification in future studies. Myocardial edema cannot be validated by classical histology, as it is complicated by dehydration through tissue fixation with formaldehyde. However, a previous experimental study used a tissue desiccation method to provide validation of T2 mapping measurements against myocardial water content [37]. CMR examinations in the present study were performed as a part of clinical service, where the much-needed time efficiency does not justify the use of rest perfusion imaging nor postcontrast T1 mapping.

## Conclusions

In conclusion, we demonstrate independent associations between serological biomarkers with imaging markers of interstitial expansion, which are CKD-group specific. Our findings indicate the role of diffuse non-ischemic tissue processes, including excess of myocardial fluid in addition to diffuse fibrosis in CKD-related adverse remodeling.

## Supplementary Information

**Additional file 1:** **Figure S1:** Heat map for correlations between mapping parameters with clinical and CMR findings at different CKD stages. A more intense color indicates closer association (blue for direct correlation, red for inverse correlation). NA (not applicable), eGFR—estimated glomerular filtration rate (in units, ml/min/1.73 m^2^), CRP—C-reactive protein, EDV—end-diastolic volume, ESV—end-systolic volume, EF—ejection fraction, GLS—global longitudinal strain, LV—left ventricular, LGE—late gadolinium enhancement, IVC—inferior vena cava.

**Additional file 2: Figure S2:** Heat map for correlations between cardiac biomarkers and CMR imaging markers of adverse cardiac remodeling at different CKD stages. A more intense color indicates closer association (blue for direct correlation, red for inverse correlation). eGFR—estimated glomerular filtration rate (in units, ml/min/1.73 m^2^), EV—end-diastolic volume, ESV—end-systolic volume, EF—ejection fraction, GLS—global longitudinal strain, LV—left ventricular, LGE—late gadolinium enhancement, IVC—inferior vena cava.

**Additional file 3:** Supplementary methods.

## Data Availability

None.

## Abbreviations

BMI: Body mass index
BP: Blood pressure
CAD: Coronary artery disease
CKD: Chronic kidney disease
CMR: Cardiovascular magnetic resonance
CRP: C-reactive protein
CVD: Cardiovascular disease
eGFR: Estimated glomerular filtration rate
GBCA: Gadolinium-based contrast agent
GCS: Global circumferential strain
GLS: Global longitudinal strain
HF: Heart failure
hs-cTnT: Cardiac biomarkers troponin T
IVC: Inferior vena cava
IQR: Interquartile range
LA: Left atrium
LGE: Late gadolinium enhancement
LV: Left ventricle/left ventricular
LVEDV: Left ventricular end-diastolic volume
LVEF: Left ventricular ejection fraction
LVESV: Left ventricular end-systolic volume
LVH: Left ventricular hypertrophy
MDRD: Modification of Diet in Renal Disease
MOLLI: Modified Look-Locker inversion recovery
NSF: Nephrogenic systemic fibrosis
NT-pro BNP: NT-pro-brain nucleotide peptide
PSM: Propensity score matching
RVEF: Right ventricular ejection fraction
SD: Standard deviation
SE: Standard error
