# Supplementary figures and images for: Cardiac biomarkers in chronic kidney disease are independently associated with myocardial edema and diffuse fibrosis by cardiovascular magnetic resonance
Source: J Cardiovasc Magn Reson. 2021 Jun 7;23:71. doi: 10.1186/s12968-021-00762-z (PMC8183054; doi:10.1186/s12968-021-00762-z)

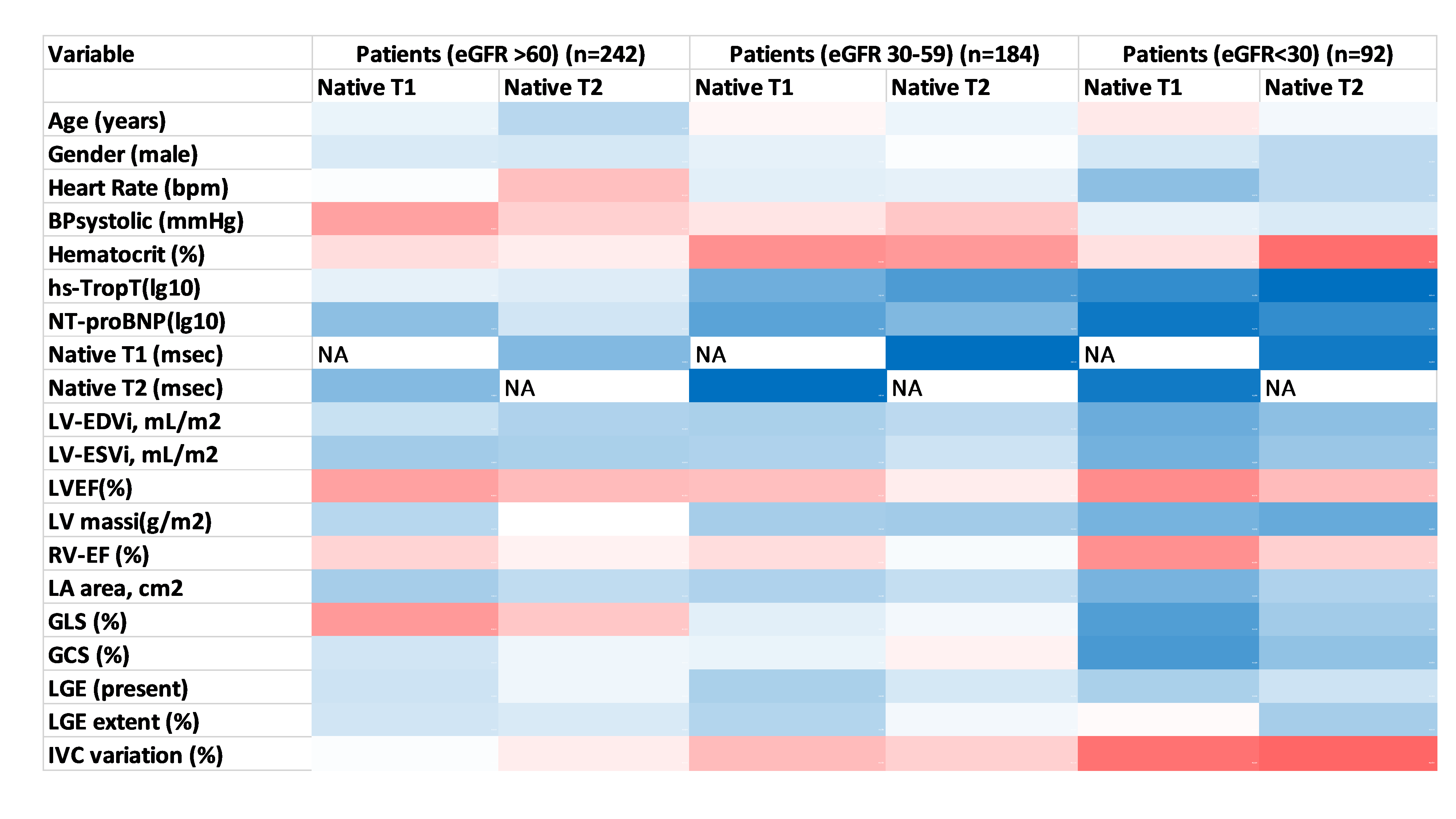

Supplement: Supplementary file 1 — Additional file 1: Figure S1: Heat map for correlations between mapping parameters with clinical and CMR findings at different CKD stages. A more intense color indicates closer association (blue for direct correlation, red for inverse correlation). NA (not applicable), eGFR—estimated glomerular filtration rate (in units, ml/min/1.73 m2), CRP—C-reactive protein, EDV—end-diastolic volume, ESV—end-systolic volume, EF—ejection fraction, GLS—global longitudinal strain, LV—left ventricular, LGE—late gadolinium enhancement, IVC—inferior vena cava. [file 12968_2021_762_MOESM1_ESM.tif]

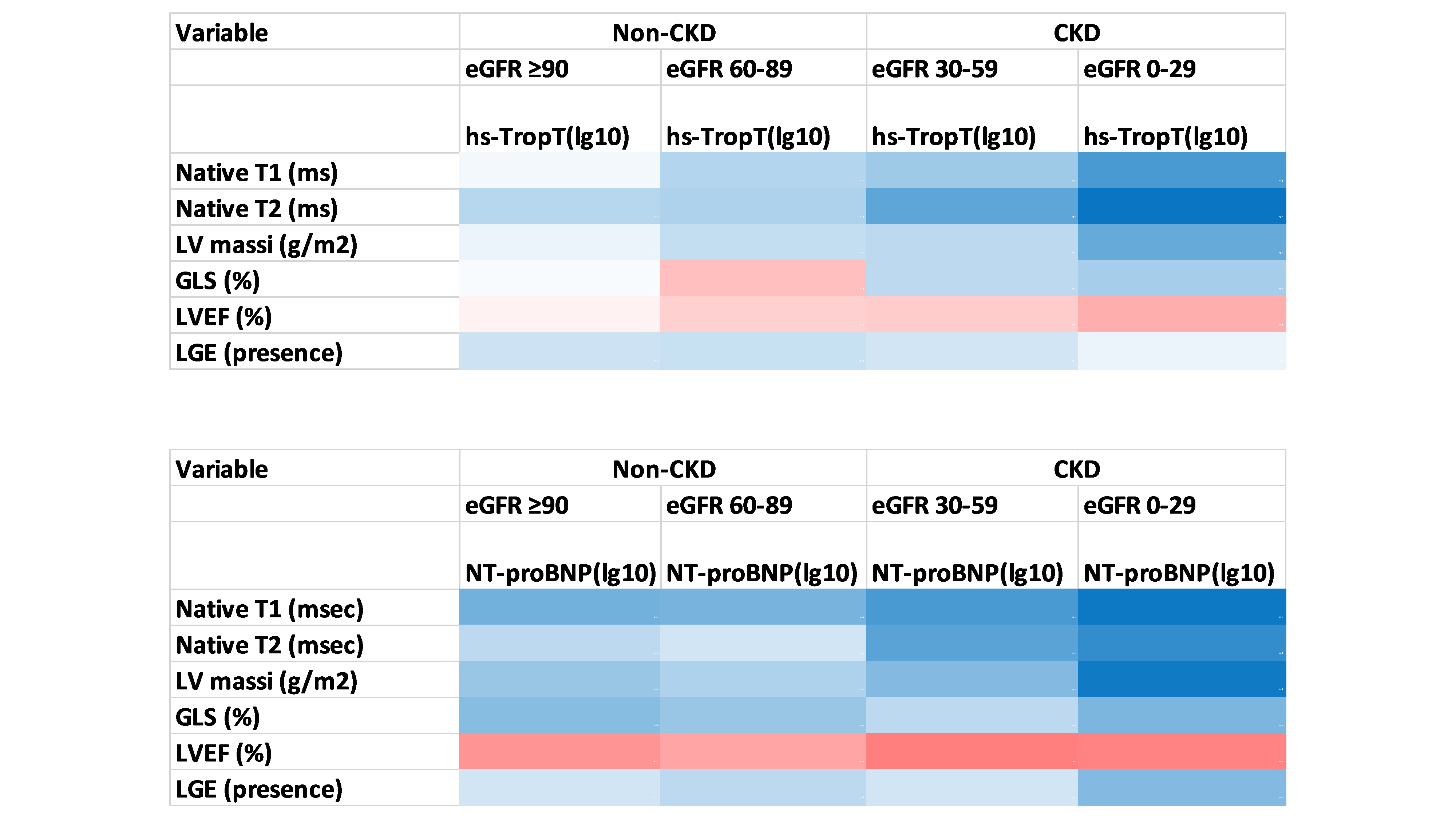

Supplement: Supplementary file 2 — Additional file 2: Figure S2: Heat map for correlations between cardiac biomarkers and CMR imaging markers of adverse cardiac remodeling at different CKD stages. A more intense color indicates closer association (blue for direct correlation, red for inverse correlation). eGFR—estimated glomerular filtration rate (in units, ml/min/1.73 m2), EV—end-diastolic volume, ESV—end-systolic volume, EF—ejection fraction, GLS—global longitudinal strain, LV—left ventricular, LGE—late gadolinium enhancement, IVC—inferior vena cava. [file 12968_2021_762_MOESM2_ESM.tif]
