# Supplementary material for: Cardiac biomarkers in chronic kidney disease are independently associated with myocardial edema and diffuse fibrosis by cardiovascular magnetic resonance
Source: J Cardiovasc Magn Reson. 2021 Jun 7;23:71. doi: 10.1186/s12968-021-00762-z (PMC8183054; doi:10.1186/s12968-021-00762-z)
Supplement: Supplementary file 3 — Additional file 3: Supplementary methods. [file 12968_2021_762_MOESM3_ESM.docx]

**Supplementary material**

**Patients’ enrollment and registries:**

All patients underwent CMR examination after clinical referral, i.e defined by a guideline-based clinical indication for CMR. Based on a formal CKD diagnosis, as made by a nephrologist based, patients were included within the TrueType CKD rather than T1 Outcome registry. The imaging protocols and parameters of these two studies are identical, as such clinical research procedures and the imaging studies were comparable, as described below in the section “sequence parameters”. Though technically identical, inclusion of CKD patients within a separate registry was made in order to provide for a specific ethic approval in this subset of patients, in relation to the additional, albeit optional, post-dialysis study. Both study protocols are described in their respective NCT submission [NCT03749551](https://clinicaltrials.gov/ct2/show/NCT03749551) and [NCT03749343](https://clinicaltrials.gov/ct2/show/NCT03749343) respectively.

**Sequence parameters:**

1. All examinations were performed on a 3-Tesla clinical scanner (Skyra, Siemens Healthineers, Erlangen, Germany, Software Version VE11)
2. Cine imaging was performed using a balanced steady-state free precession sequence in combination with parallel imaging (SENSitivity Encoding, factor 2) and retrospective gating during expiratory breath- hold (TE/TR/flip-angle: 1.7ms/3.4ms/60°, spatial resolution 1.8x1.8x8 mm), as a gapless short axis (SAX) stack for assessment of cardiac volumes and function, or single slice long-axis views (2-chamber, 3-chamber, and 4 chamber view).
3. Myocardial perfusion was performed using dynamic saturation recovery first pass of a bolus of 0.1 mmol/kg body weight gadobutrol (Gadovist®, Bayer AG, Germany) after administration of Regadenoson bolus (400mcg/4ml iv.). Because the analysis of perfusion images was visual (see below), rest perfusion imaging was not employed, to reduce the amount of contrast agent and the overall table-time.
4. LGE imaging was performed using gapless whole heart coverage of SAX slices ~15 minutes after administration of above bolus of gadobutrol (Gadovist®, Bayer, Leverkusen, Germany), using a mid-diastolic inversion prepared 2-dimensional gradient echo sequence (TE/TR/flip-angle 2.0 msec/3.4 msec/25°, acquired voxel size 1.4x1.4x8mm) with an individually adapted prepulse delay to achieve optimally nulled myocardium.
5. T1 mapping: Balanced steady state free precession single breath-hold modified Look-Locker Imaging (FFM-MOLLI) was used for T1 mapping and performed in a single midventricular SAX slice at mid-diastole, prior to contrast administration, respectively (TE/TR/flip-angle: 1.64msec/3.3msec/50°, acquired voxel size 1.8x1.8x8 mm, phase encoding steps n=166, 6/8 half scan, 11 images corresponding to 3 different inversion times using a nonselective 180° prepulse in an algorithm of 3’3’5 MOLLI scheme (1). T1 mapping sequence exam cards used in this study have been published previously(1).
6. T2-prepared SSFP pulse sequence (T2-FLASH): (2) A T2-prepared steady-state free precession sequence was used to generate three T2W images, one each with different T2 preparation times (TE T2P = 0 ms, 24 ms, 55 ms). Other sequence parameters include repetition time 3 × RR, acquisition time 7xRR, image matrix 96 × 160, echo spacing (ms) 2.6, single shot acquisition, flip angle 40°, bandwidth 1488, parallel acquisition (acceleration) GRAPPA 2.

**Transthoracic echocardiography**

Transthoracic echocardiography was performed by board-certified cardiologists (LA, HaZ, MG, VP) within 30 minutes of completion of CMR examination with participants lying supine in left lateral position (Vivid E95, GE Healthcare, Chicago, IL) for respiratory variation of inferior vena cava (IVC) using postprocessing recommendation on workstation (EchoPAC, GE Healthcare, Chicago, IL) (3).

Normal values

We established the above MOLLI sequence on Philips platform (Intera, Achieva, software version 4) in 2010 and continued using it till the end of 2014. This sequence is no longer supported on Philips after release 5). In 2015 we reverse-engineered this sequence and transferred it over to the Siemens platforms (software version V11E). We validated the Siemens derived values in phantoms, traveling healthy volunteers and a few thousands of patients. We also obtained histological and outcome data on both vendors. As such we are reassured that the original normal values and SD remain sufficiently similar to be cross-applicable. Hence, the citations provided refer to the exact sequence parameters, even if the current data collection was performed on the Siemens platform.

1. Puntmann VO, Carr-White G, Jabbour A et al. T1-Mapping and Outcome in Nonischemic Cardiomyopathy. JACC. Cardiovascular imaging 2016; 9: 40–50.

2. Giri S, Chung Y-C, Merchant A et al. T2 quantification for improved detection of myocardial edema. Journal of Cardiovascular Magnetic Resonance 2009; 11: 56.

3. Galderisi M, Cosyns B, Edvardsen T et al. Standardization of adult transthoracic echocardiography reporting in agreement with recent chamber quantification, diastolic function, and heart valve disease recommendations: an expert consensus document of the European Association of Cardiovascular Imaging. European Heart Journal - Cardiovascular Imaging 2017; 18: 1301–1310.
